# Oxygen radical based on non-thermal atmospheric pressure plasma alleviates lignin-derived phenolic toxicity in yeast

**DOI:** 10.1186/s13068-020-1655-9

**Published:** 2020-01-28

**Authors:** Shou Ito, Kiyota Sakai, Vladislav Gamaleev, Masafumi Ito, Masaru Hori, Masashi Kato, Motoyuki Shimizu

**Affiliations:** 1 Faculty of Agriculture, Meijo University, Nagoya, Aichi 468-8502 Japan; 2 Faculty of Science and Technology, Meijo University, Nagoya, Aichi 468-8502 Japan; 3 Center for Low-temperature Plasma Sciences, Nagoya University, Nagoya, Aichi 464-8603 Japan

**Keywords:** Atmospheric pressure plasma, Oxygen-radical treatment, Biorefinery, Bioethanol, Plant biomass, Vanillin

## Abstract

**Background:**

Vanillin is the main byproduct of alkaline-pretreated lignocellulosic biomass during the process of fermentable-sugar production and a potent inhibitor of ethanol production by yeast. Yeast cells are usually exposed to vanillin during the industrial production of bioethanol from lignocellulosic biomass. Therefore, vanillin toxicity represents a major barrier to reducing the cost of bioethanol production.

**Results:**

In this study, we analysed the effects of oxygen-radical treatment on vanillin molecules. Our results showed that vanillin was converted to vanillic acid, protocatechuic aldehyde, protocatechuic acid, methoxyhydroquinone, 3,4-dihydroxy-5-methoxybenzaldehyde, trihydroxy-5-methoxybenzene, and their respective ring-cleaved products, which displayed decreased toxicity relative to vanillin and resulted in reduced vanillin-specific toxicity to yeast during ethanol fermentation. Additionally, after a 16-h incubation, the ethanol concentration in oxygen-radical-treated vanillin solution was 7.0-fold greater than that from non-treated solution, with similar results observed using alkaline-pretreated rice straw slurry with oxygen-radical treatment.

**Conclusions:**

This study analysed the effects of oxygen-radical treatment on vanillin molecules in the alkaline-pretreated rice straw slurry, thereby finding that this treatment converted vanillin to its derivatives, resulting in reduced vanillin toxicity to yeast during ethanol fermentation. These findings suggest that a combination of chemical and oxygen-radical treatment improved ethanol production using yeast cells, and that oxygen-radical treatment of plant biomass offers great promise for further improvements in bioethanol-production processes.

## Background

Biorefinement of lignocellulosic biomass to liquid fuels or other chemicals is beneficial to sustainable energy and the environment [1]. Lignocellulose mainly comprises cellulose, hemicellulose, and lignin, and cellulose and hemicellulose are capable of converting fermentable sugars by enzymatic hydrolysis, whereas lignin plays a negative role in saccharification of the lignocellulosic biomass [2]. Lignin is an aromatic polymer comprising three primary units [hydroxyphenyl (H), guaiacyl (G), and syringyl (S)] that are randomly linked with aryl ether, ester, or carbon bonds [3, 4].

Bioethanol production from lignocellulose generally involves three steps: (1) pretreatment to break down complex lignocellulose structures, (2) enzymatic hydrolysis of polysaccharides (i.e., cellulose and hemicellulose) into fermentable sugars, and (3) fermentation to convert sugars into ethanol [5]. Pretreatment is required to alter the biomass by changing its chemical or physical properties and to allow increased enzyme accessibility to cellulose [6, 7], with various biological, chemical, and physical pretreatment methods having been developed [8–12]. Vanillin is generally generated as a byproduct during the process of fermentable-sugar production from lignocellulosic biomass, regardless of being herbage, softwood, or hardwood [13, 14]. The vanillin concentration in the lignocellulosic hydrolysate can vary depending on the types of biomass materials and treatment methods, with a wide range of vanillin concentrations (1–26 mM) reported in previous studies [15, 16]. Because vanillin is a potent inhibitor of yeast-specific ethanol fermentation via dose-dependent blockage of yeast growth and subsequent fermentation, vanillin toxicity represents a major barrier to reducing the cost of bioethanol production [17–20]. Several methods, including overliming, anion-exchange resin treatment, activated carbon treatment, sulphate treatment, and treatment with laccase, have been proposed to alleviate the negative effects of lignin-derived phenolics on biomass hydrolysates [21–25]; however, these methods require long processing times and are detrimental to the environment based on the release of organic waste [21, 23]. Additionally, utilization of these methods requires alkaline- or acid-resistant equipment, a neutralization step, chemical recovery, and waste treatment [21–25]. Therefore, the development of an environmentally friendly vanillin-removal process is an important prerequisite for the efficient production of bioethanol from lignocellulosic biomass.

In our previous work, we developed radical generators based on non-thermal atmospheric pressure plasma (NTAP) technology using an available radical generator with an oxygen–argon gas mixture to generate oxygen radicals [26, 27]. The radical generator provides high electron density, and we reported large amounts of atomic –O (^3^P_*j*_) at an absolute density on the order of between 10^13^ cm^−3^ and 10^14^ cm^−3^ (equivalent to 1–10 ppm) [28]. Use of the NTAP-based radical generator has several advantages: (1) on-site generation, which avoids problems associated with chemical supply and storage; (2) reaction at ambient temperatures and pressures; (3) achievement of a rapid reaction with a high density of atomic oxygen radicals; and (4) a low cost relative to conventional low-pressure plasmas due to the absence of vacuum devices [29]. Moreover, pretreatment of plant biomass using a radical generator is more environmentally friendly than chemical methods, given that no chemical waste is produced. In our recent work, oxygen-radical pretreatment of cellulose and wheat straw enhanced cellulose degradation by cellobiohydrolases (CBHs) from the white-rot fungus *Phanerochaete chrysosporium* [30]. These findings indicated that the NTAP-based radical generator offers great promise for use in biorefining processes.

In this study, we analysed the effects of oxygen-radical irradiation against vanillin molecules, potent inhibitors of ethanol production by yeast. We also determined the effects of oxygen-radical treatment on lignin-derived phenolics generated by alkaline-pretreated rice straw.

## Results and discussion

### Oxygen-radical irradiation of vanillin

**Fig. 1:**
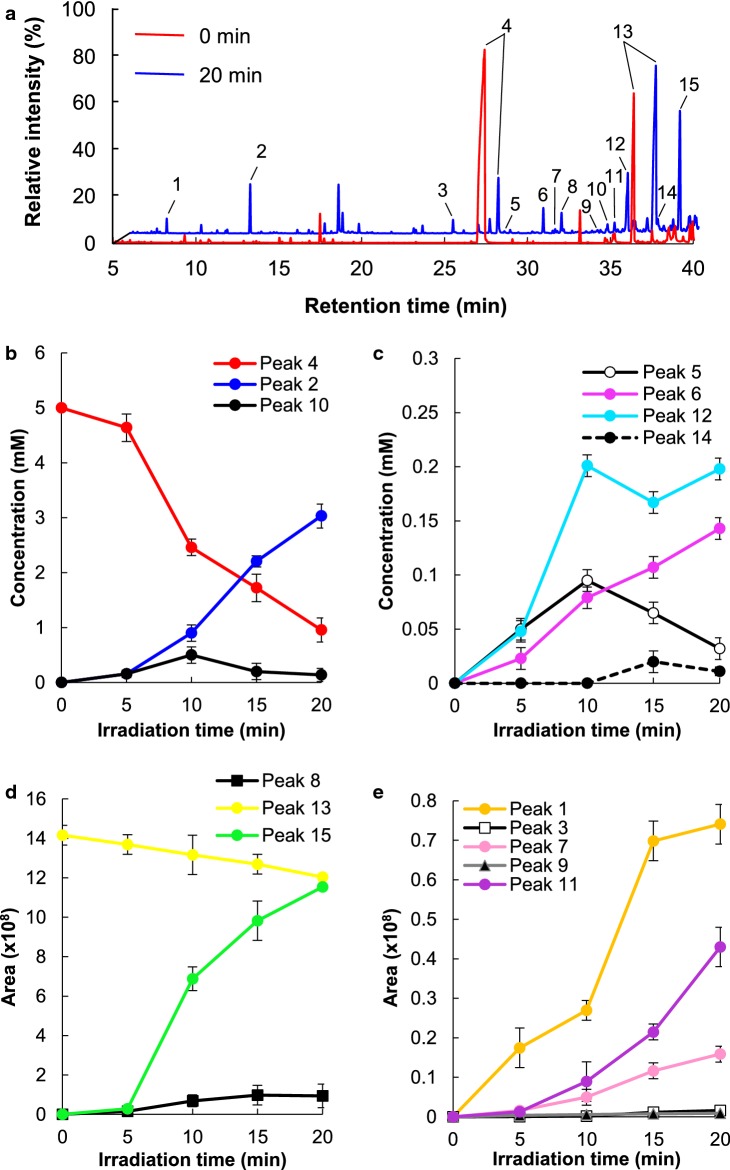
Conversion of vanillin by oxygen-radical treatment. **a** GC–MS chromatogram of vanillin solution (5.0 mM) irradiated with oxygen-radical treatment for 0 min and 20 min. Reaction products were trimethylsilylated and analysed by GC–MS. Identified reaction products are marked by arrows with numbers and shown in Table 1. **b**–**e** Treatment-time-dependent conversion of vanillin and the production of reactants. Error bars represent the mean ± standard error of the mean of three independent experiments

The effects of oxygen-radical irradiation of vanillin were examined using high-performance liquid chromatography (HPLC) and GC–MS (Fig. 1a and Additional file 1: Figure S1). Time-course analysis of vanillin conversion by oxygen-radical treatment using HPLC showed that the vanillin concentration in oxygen-radical-treated solutions decreased with increasing treatment time (Additional file 1: Figure S1). Vanillin (5.0 mM) decreased to 0.96 mM and was converted to vanillic acid (0.20 mM), protocatechuic aldehyde (0.14 mM), protocatechuic acid (0.01 mM), methoxyhydroquinone (0.03 mM), 3,4-dihydroxy-5-methoxybenzaldehyde (0.14 mM), and trihydroxy-5-methoxybenzene by oxygen-radical irradiation for 20 min using the radical generator (Fig. 1 and Additional file 1: Figure S2; Table 1). Additionally, we detected aromatic-ring-cleaved products, including methyl-2,5-dihydroxy-6-oxohexa-2,4-dienoate, 4-hydroxy-6-methoxy-6-oxohexa-2,4-dienoic acid, 4-formyl-6-methoxy-6-oxohexa-2,4-dienoic acid, 4-(2-methoxy-2-oxoethylidene)pent-2-enedioic acid, oxalic acid (3.03 mM), and methoxy oxalic acid, indicating that the benzene-ring of vanillin and its derivatives were cleaved by oxygen-radical irradiation. Moreover, we detected an unidentified but putative aromatic dimer compound (Fig. 1 and Additional file 1: Figure S2; Table 1). These results suggested that oxygen-radical irradiation promoted vanillin oxidation, monooxygenation, demethoxylation, decarbonylation, dimerization, and aromatic-ring fission (Additional file 1: Figure S3).

**Table 1:** Detected vanillin-specific compounds derived from oxygen-radical treatment

No.	Identified compounds	Concentration (mM)
1	Methoxy oxalic acid	–^a^
2	Oxalic acid	3.031 ± 0.618
3	Methyl-2,5-dihydroxy-6-oxohexa-2,4-dienoate	–
4	Vanillin	0.957 ± 0.133
5	Methoxyhydroquinone	0.032 ± 0.008
6	Protocatechuic aldehyde	0.143 ± 0.043
7	4-Hydroxy-6-methoxy-6-oxohexa-2,4-dienoic acid	–
8	4-Formyl-6-methoxy-6-oxohexa-2,4-dienoic acid	–
9	Trihydroxy-5-methoxybenzene	–
10	3,4-Dihydroxy-5-methoxybenzaldehyde	0.139 ± 0.037
11	4-(2-Methoxy-2-oxoethylidene)pent-2-enedioic acid	–
12	Vanillic acid	0.198 ± 0.050
13	Unidentified compound that is contaminant in original reagent	–
14	Protocatechuic acid	0.011 ± 0.003
15	Unidentified compound that is putative aromatic dimer	–

Initial concentration of vanillin was 5.0 mM. The concentrations of vanillin and the reactants irradiated with oxygen-radical treatment for 20 min were quantified by GC–MS. Reaction products were trimethylsilylated and analysed by GC–MS. Numbers indicate the GC peaks shown in Fig. 1a. Mass spectra obtained from each GC peak are shown in Additional file 1: Figure S2

^a^These compounds were not quantified because these reagents were not commercially available

Previous studies indicated that the molecular weights of amino acids, such as Tyr, Phe, Trp, Cys, Met, Pro, His, Lys, Arg, Gln, Glu, Val, Leu, and Ile, change due to oxidation and hydroxylation by active species generated by NTAP irradiation [31–33]. Specifically, electron-rich groups, such as nitrogen- and sulphur-containing and aromatic compounds, were preferentially modified by the various active species [31–33]. Additionally, the aromatic rings of Tyr, Phe, Trp, and His are reportedly hydroxylated by NTAP irradiation [32]. Using Fourier transform and ^1^H nuclear magnetic resonance analysis, Asandulesa et al. [34] showed that the aromatic rings of benzyl alcohol, benzaldehyde, and benzyl chloride were cleaved and converted to aliphatic groups by NTAP irradiation. Moreover, similar results were observed using pyrolytic lignin and phenolic model compounds by ozonolysis [35–37]. Although the exact mechanism of vanillin conversion and aromatic-ring cleavage by oxygen-radical, plasma, or ozone treatment is not fully elucidated, oxygen-radical treatment would likely generate radicals in the gas phase that would react with lignin-derived phenolics to form radicals that promote ring cleavage. These findings indicated that vanillin oxidation, monooxygenation, demethoxylation, decarbonylation, dimerization, and aromatic-ring fission were generated by oxygen-radical treatment (Additional file 1: Figure S3).

### Effects of oxygen-radical treatment on yeast growth and ethanol production

**Fig. 2:**
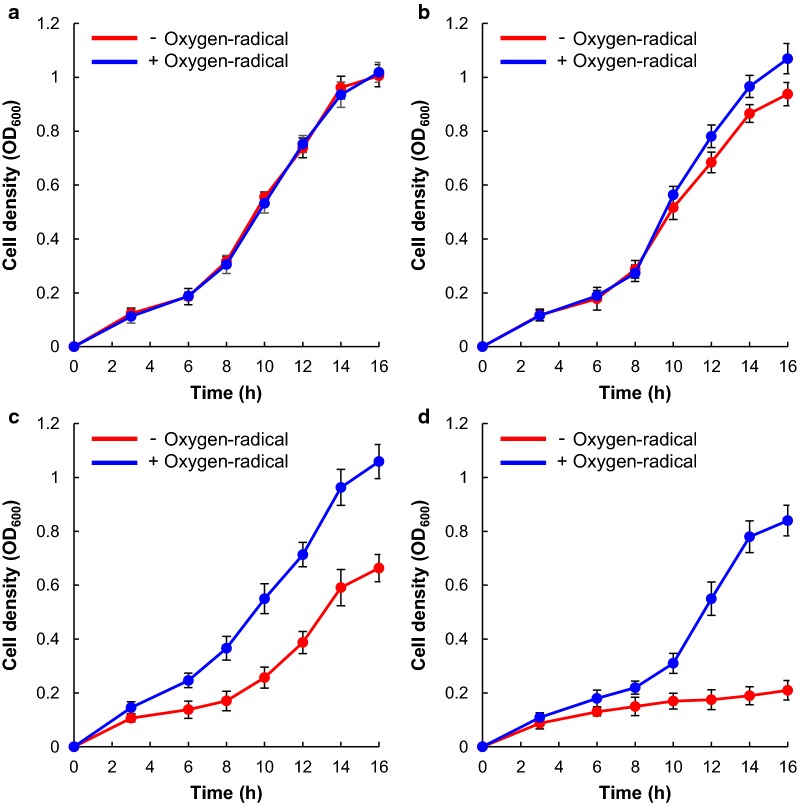
Effects of oxygen-radical treatment of vanillin on the growth of *S. cerevisiae*. The yeast was grown in YPD medium supplemented with **a** 0 mM, **b** 1 mM, **c** 2.5 mM, and **d** 5.0 mM vanillin with or without oxygen-radical treatment. Yeast growth was monitored by measuring optical density at 600 nm. Error bars represent the mean ± standard error of the mean of three independent experiments

**Fig. 3:**
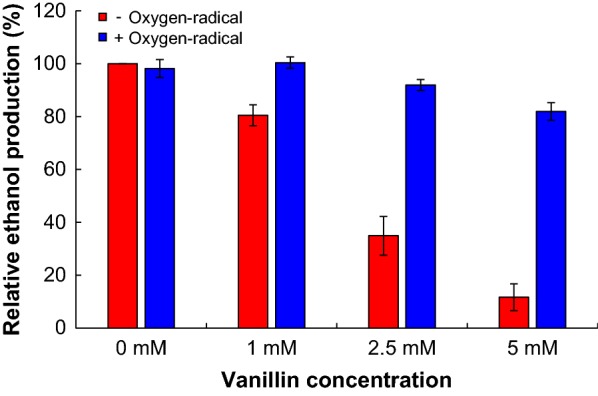
Effects of oxygen-radical treatment of vanillin on ethanol production by *S. cerevisiae*. Yeast was grown in YPD medium supplemented with 0 mM, 1 mM, 2.5 mM, and 5.0 mM vanillin with or without oxygen-radical treatment. After a 16-h incubation, ethanol in the culture supernatant was measured. The ethanol production by *S. cerevisiae* in YPD medium supplemented with 0 mM vanillin without oxygen-radical treatment was set at 100%. Error bars represent the mean ± standard error of the mean of three independent experiments

To examine the effects of oxygen-radical treatment of vanillin solution on yeast growth, we cultivated *Saccharomyces cerevisiae* S288c in YPD medium containing up to 5 mM vanillin irradiated with or without oxygen-radical. Figure 2 shows the yeast-growth curves associated with various vanillin concentrations. Compared with the absence of vanillin, yeast growth was inhibited by 8%, 35%, and 80% in the presence of 1.0 mM, 2.5 mM, and 5.0 mM vanillin, respectively, whereas the growth rates were 105%, 104%, and 83% in the presence of vanillin irradiated with oxygen-radical, respectively (Fig. 2a–d). The effect of several vanillin degradation products, such as vanillic acid, protocatechuic aldehyde, protocatechuic acid, methoxyhydroquinone, 3,4-dihydroxy-5-methoxybenzaldehyde, and oxalic acid on yeast growth was also determined (Additional file 1: Figure S4). Yeast growth with 2.5 mM vanillin was inhibited the most compared with that with the same concentration of its degradation products. These results indicate that vanillin degradation products generated by oxygen-radical treatment have lower toxicity against *S. cerevisiae* cells. The concentrations of vanillin degradation products except oxalic acid were lower than that of vanillin (Fig. 1 and Additional file 1: Figure S2; Table 1). Yeast growth was inhibited by 15% in the presence of 2.5 mM oxalic acid (Additional file 1: Figure S4). Compared with the absence of vanillin, yeast growth was inhibited by 8% in the presence of 1.0 mM vanillin, whereas the growth rate was 83% in the presence of 5.0 mM vanillin irradiated with oxygen-radical for 20 min, respectively (Fig. 2b, d). These results suggest that yeast growth in the presence of 5.0 mM vanillin irradiated with oxygen-radical may be inhibited by 20% by residual vanillin (0.96 mM) and oxalic acid (3.03 mM) generated from vanillin by oxygen-radical treatment (Fig. 2d). Moreover, ethanol concentration in culture supernatant after 16-h incubation in the absence of vanillin was 10.4 g/L (Fig. 3), whereas inclusion of vanillin inhibited ethanol production by 20%, 66%, and 88% at 1.0 mM, 2.5 mM, and 5.0 mM vanillin, respectively. Compared with the 16 h incubation in the absence of vanillin, ethanol production was 100%, 92%, and 83% in the presence of 1.0 mM, 2.5 mM, and 5.0 mM vanillin irradiated with oxygen radical, respectively (Fig. 3). The ethanol concentration in the oxygen-radical-treated vanillin solution at 5.0 mM was 7.0-fold greater than that from non-treated solution (Fig. 3). These results suggested that irradiation with oxygen radical alleviated vanillin toxicity against *S. cerevisiae* and helped to restore 80% of the ethanol yield as compared with no vanillin present.

Vanillin acts as a potent fermentation inhibitor that represses yeast growth and fermentative abilities [20, 38]. A recent study showed that vanillin suppressed translation initiation by affecting the ribosome-assembly process, thereby causing accumulation of cytoplasmic messenger ribonucleoprotein granules and processing bodies [39]. Furthermore, vanillin induces the accumulation of reactive oxygen species and mitochondrial fragmentation in *S. cerevisiae* and limits mRNA translation to reduce overall protein-synthesis levels, leading to vanillin-specific inhibition of yeast cell growth and ethanol fermentation [40, 41]. *S. cerevisiae* is a traditionally competitive cell factory used for bioethanol production due to its superior tolerance to ethanol and low pH, as well as its ease of genetic manipulation [42]. To overcome vanillin toxicity as a barrier to reduced bioethanol-production costs, vanillin-tolerant strains have been screened and engineered [38, 43–45]; however, these strains have not fully resolved the problems of toxicity associated with lignin-derived phenolics, which have been documented in other fermentable microorganisms (i.e., ethanol fermentation by *Thermoanaerobacter mathranii*, butanol fermentation by *Clostridium beijerinckii* and *Clostridium acetobutylicum*, butyric acid fermentation by *Clostridium tyrobutyricum*, hydrogen fermentation by *Thermoanaerobacter thermosaccharolyticum*, bacterial nanocellulose production by *Gluconacetobacter xylinus*, and xylitol fermentation of *Candida tropicalis*) [46–52]. Therefore, the presence of lignin-derived phenolics remains a problem in biorefining processes using lignocellulosic biomass. Our results suggest that oxygen-radical treatment as a potentially effective means of addressing vanillin toxicity to microorganisms during biorefining processes.

### Effects of oxygen-radical treatment on lignin-derived phenolics generated by alkaline pretreatment of plant biomass

We examined the effects of oxygen-radical treatment of alkaline-pretreated rice straw slurry on yeast growth and ethanol production. The composition of cellulose, hemicellulose, lignin, ash, and total solids in non-pretreated rice straw and alkaline-pretreated rice straw with or without oxygen-radical treatment was determined (Table 2). After alkaline pretreatment, the biomass loss of native rice straw was 31.1% (Table 2). The remaining solid of alkaline-pretreated rice straw without oxygen-radical treatment was 68.9%, including 65.4% cellulose, 18.2% hemicellulose, 5.5% lignin, and 5.1% ash (Table 2). Oxygen-radical treatment did not affect the composition of alkaline-pretreated rice straw (Table 2).

**Table 2:** The content of cellulose, hemicellulose, lignin, and ash in native, alkaline-pretreated and alkaline-pretreated with oxygen-radical-treated rice straw

Treatment		Total solids^a^ (%)	Composition (%)				Remaining solid (%)
Alkaline	Oxygen-radical		Cellulose	Hemicellulose	Lignin	Ash	
−	−	96.1	34.4	29.5	13.4	13.1	–
+	−	98.1	65.4	18.2	5.5	5.1	68.9
+	+	97.7	63.9	18.5	5.9	5.4	67.8

The contents of cellulose, hemicellulose, lignin, and ash in native and alkaline-pretreated rice straw with or without oxygen-radical treatment were determined according to previous methods. Data are presented as mean values of three independent experiments. The standard errors were < 22%

^a^Based on dry matter

**Fig. 4:**
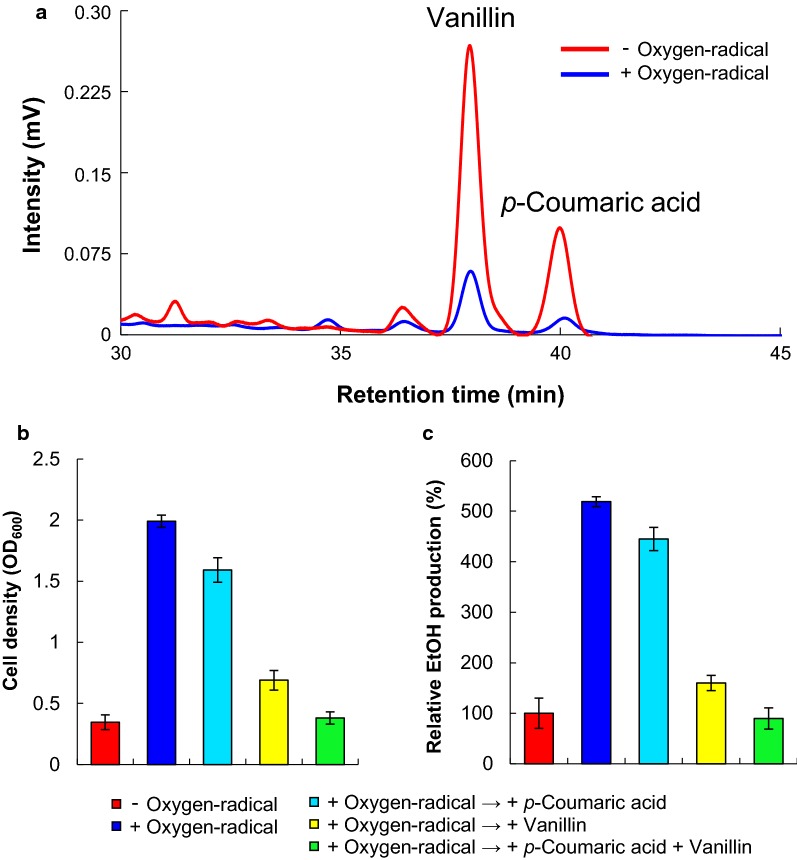
Synergistic action of alkaline-pretreatment and oxygen-radical treatment on lignin-derived phenolics generated from rice straw. **a** HPLC chromatogram of vanillin and *p*-coumaric acid in the alkaline-pretreated rice straw slurry with or without oxygen-radical treatment for 20 min. Peaks of vanillin and *p*-coumaric acid at retention times of 38.0 min and 39.8 min, respectively. Effects of oxygen-radical treatment of the alkaline-pretreated rice straw slurry on the **b** growth and **c** ethanol production of *S. cerevisiae*. Vanillin and/or *p*-coumaric acid were added to the oxygen-radical-treated suspensions at final concentrations of 3.3 mM and/or 2.1 mM, respectively, followed by determination of yeast growth and ethanol production. Error bars represent the mean ± standard error of the mean of three independent experiments

We then performed vanillin conversion in the alkaline-pretreated rice straw slurry following oxygen-radical treatment for 20 min using HPLC (Fig. 4a) and GC–MS. Analysis of the soluble products from alkaline-treated rice straw revealed vanillin (3.32 mM), vanillic acid (0.13 mM), *p*-coumaric acid (2.11 mM), *t*-ferulic acid (0.69 mM), oxalic acid (1.13 mM), lactic acid (0.50 mM), furfural (0.02 mM), and HMF (0.01 mM) (Table 3). These results indicated that lignin in native rice straw was converted to vanillin (7.5%), vanillic acid (0.3%), *p*-coumaric acid (5.2%), and *t*-ferulic acid (2.0%) in the alkaline-treated rice straw slurry without oxygen-radical treatment (Tables 2 and 3). However, vanillin concentration in the oxygen-radical-treated slurry decreased to 0.69 mM (Fig. 4a; Table 3). Additionally, *p*-coumaric acid, a potent inhibitor of yeast growth [53], was decreased to 0.31 mM in the oxygen-radical treated slurry (Table 3). Although yeast growths with 2.5 mM *p*-coumaric acid, oxalic acid, lactic acid, and furfural were 1.59-, 1.61-, 1.62-, and 1.60-fold, higher, respectively, than that with 2.5 mM vanillin, the compounds inhibited yeast growth (Additional file 1: Figures S4 and S5). These results implied that vanillin conversion by the oxygen-radical treatment of alkaline-pretreated rice straw enhanced yeast growth and ethanol production.

**Table 3:** Detected compounds in alkaline-pretreated rice straw slurry with or without oxygen-radical treatment

No.	Identified compounds	Concentration (mM)	
		Without oxygen-radical	With oxygen-radical
1	Methoxy oxalic acid	ND	–^a^
2	Oxalic acid	1.130 ± 0.232	2.311 ± 0.329
3	Vanillin	3.320 ± 0.541	0.691 ± 0.144
4	Methoxyhydroquinone	ND	0.082 ± 0.029
5	Protocatechuic aldehyde	ND	0.077 ± 0.023
6	4-Hydroxy-6-methoxy-6-oxohexa-2,4-dienoic acid	ND	–
7	3,4-Dihydroxy-5-methoxybenzaldehyde	ND	0.096 ± 0.030
8	4-(2-Methoxy-2-oxoethylidene)pent-2-enedioic acid	ND	–
9	Vanillic acid	0.134 ± 0.052	0.055 ± 0.020
10	Lactic acid	0.502 ± 0.132	0.991 ± 0.203
11	Furfural	0.021 ± 0.003	0.011 ± 0.003
12	HMF	0.010 ± 0.003	ND
13	*p*-Coumaric acid	2.105 ± 0.478	0.314 ± 0.090
14	*t*-Ferulic acid	0.687 ± 0.233	0.223 ± 0.044

The concentrations of several compounds detected in alkaline-pretreated rice straw slurry with or without oxygen-radical treatment were quantified by GC–MS. These were trimethylsilylated and analysed by GC–MS

*ND* not detected

^a^These compounds were not quantified because these reagents were not commercially available

We then performed cellulase, from *Aspergillus niger*, hydrolysis of alkaline-pretreated rice straw slurry with or without oxygen-radical treatment to produce fermentable sugars to promote ethanol production by yeast. Following enzymatic hydrolysis, we analysed the soluble products in the alkaline-treated rice straw suspensions with or without oxygen-radical treatment by reducing-sugar HPLC, finding that the contents of reducing sugars, such as glucose, cellobiose, cellotriose, and xylose were similar regardless of oxygen-radical treatment (Additional file 1: Figure S6). Cellulose to glucose conversion rates in the alkaline-treated rice straw slurry with or without oxygen-radical and cellulase treatments were 31.0% and 32.7%, respectively. Commercially available cellulase from *A. niger* used in this study was not inhibited by up to 10 mM vanillin (data not shown).

We also determined the effect of irradiation of glucose (Additional file 1: Figure S7a). Glucose solutions (10, 25, 50 mM) were prepared, and the oxygen-radical was irradiated in these solutions. Glucose was not converted by the oxygen-radical treatment (Additional file 1: Figure S7a). We then determined yeast growth in 50 mM glucose solution with or without oxygen-radical treatment for 20 min (Additional file 1: Figure S7b). Compared with the glucose solution without oxygen-radical treatment, yeast growth was similar in oxygen-radical-treated solution (Additional file 1: Figure S7b). These results indicate that the irradiation of glucose is not affected on yeast growth. Our previous study reported that cleavage of the β-1,4-glycoside linkages in the cellulose backbone into smaller chains by oxygen-radical treatment promotes cellulose hydrolysis by allowing CBHs [30]. Because *A. niger* mainly secretes *endo*-β-1,4-glucanase and β-1,4-glucosidase and displays low levels of CBH production [54, 55], oxygen-radical treatment did not affect reducing-sugar production.

We then determined yeast growth in suspensions treated with oxygen radical for 20 min (Fig. 4b). After a 48-h incubation, yeast growth in oxygen-radical-treated suspensions was 5.8-fold higher than that of untreated suspensions (Fig. 4b). Furthermore, ethanol production from oxygen-radical-treated suspensions showed a 5.2-fold increase relative to that from untreated suspensions (Fig. 4c).

To elucidate the inhibitory effect of vanillin and *p*-coumaric acid in alkaline-pretreated rice straw suspensions, vanillin and *p*-coumaric acid were added to the oxygen-radical-treated suspension at final concentrations of 3.3 mM and 2.1 mM, respectively, followed by the determination of yeast growth and ethanol production, which revealed similar results to those obtained using alkaline-pretreated rice straw suspensions without oxygen-radical treatment (Fig. 4b, c). Compared with the addition of vanillin and *p*-coumaric acid, yeast growth rates in the suspensions were 1.8- or 4.6-fold in the presence of vanillin or *p*-coumaric acid at final concentrations of 3.3 mM or 2.1 mM, respectively (Fig. 4b). These results suggest that vanillin and *p*-coumaric acid conversions by oxygen-radical treatment of alkaline-pretreated plant biomass promote yeast ethanol production.

Because lignin-degradation products, such as vanillin, inhibit the cellulase activity of CBHs, oxygen-radical treatment of alkaline-pretreated rice straw represents an effective method for biorefining processes using cellulolytic enzymes [56, 57]. These findings indicated that oxygen-radical treatment not only promoted cellulose degradation by CBHs, but also improved yeast ethanol production via conversion of inhibitors, such as vanillin, produced from plant biomass.

**Fig. 5:**
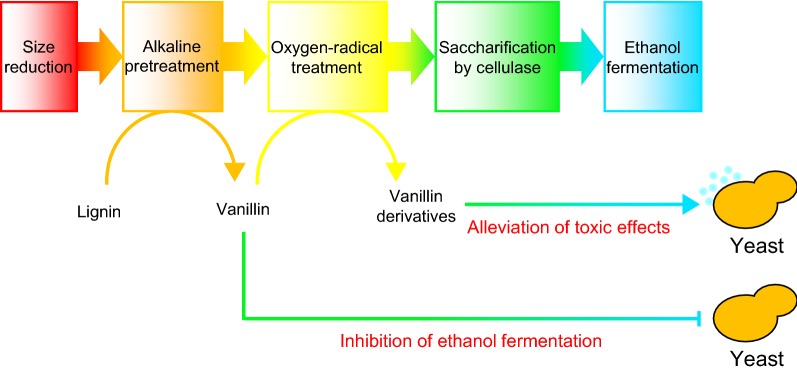
Oxygen radical treatment alleviates lignin-derived phenolic toxicity in yeast

Various biological, chemical, and physical pretreatment methods have been developed [8–12]. For economic reasons, alkaline hydrolysis is commonly used to prepare lignocelluloses for enzymatic saccharification and fermentation [58]; however, vanillin is generated as a toxic byproduct during this process [13, 14]. Yeast cells are usually exposed simultaneously to vanillin during the industrial production of bioethanol from lignocellulosic biomass. According to our findings, a combination of chemical and oxygen-radical treatment methods would improve ethanol production using yeast cells (Fig. 5). Plasma discharge generated electrically might represent an attractive treatment process for the conversion of plant biomass to ethanol.

## Conclusions

This study analysed the effects of oxygen-radical treatment on vanillin molecules, finding that this treatment converted vanillin to its derivatives, resulting in reduced vanillin toxicity to yeast during ethanol fermentation. Our results show that the oxygen-radical treatment of alkaline-pretreated lignocellulosic biomass reduces the yeast-inhibitory effects of vanillin by decreasing vanillin content while increasing the levels of various vanillin-derived molecules, thereby attenuating the inhibition of yeast growth and promoting ~ fivefold higher levels of ethanol production relative to alkaline-pretreated lignocellulosic biomass without oxygen-radical treatment. These findings suggest that the oxygen-radical treatment of plant biomass offers great promise for further improvements in bioethanol-production processes.

## Methods

### Chemicals and materials

Vanillin, vanillic acid, 3.4-dihydroxy-5-methoxybenzaldehyde (Wako Pure Chemical Industries, Osaka, Japan), 2-methoxyhydroquinone (Tokyo Chemical Industry Co., Ltd., Tokyo, Japan), protocatechuic aldehyde (Sigma-Aldrich, St. Louis, MO, USA), and protocatechuic aldehyde (Nacalai Tesque, Kyoto, Japan) were purchased and used as inhibitors of yeast growth in cultures. Cellulase (mainly containing *endo*-β-1,4-glucanase and β-1,4-glucosidase) from *A. niger* [54, 55] was obtained from Tokyo Chemical Industry Co., Ltd., and its activity was 29,500 unit/g. Rice straw was grown and harvested on the farm at Meijo University (Aichi, Japan). The straw was cut, dried at 45 °C for 3 h, and milled to a particle size of 1 mm, followed by washing at a weight ratio of 1:20 of rice straw to distilled deionized water. The washed straw was dried at 45 °C for 24 h and used for subsequent experiments.

### Oxygen-radical treatment

The oxygen-radical generator used in this study was based on an atmospheric pressure-discharge plasma generated with a gas mixture containing a small amount of O_2_ (30 sccm) in argon (4.97 slm). The use of large amounts of argon provides a high electron density on the order of 10^16^ cm^−3^ [27]. Additionally, we expected that the use of argon as a buffer would decrease the three-body collision between oxygen species resulting in O_2_ and O_3_ molecules, thereby increasing atomic oxygen production in the atmosphere. The structure of the slit with a bent-flow channel downstream is capable of intercepting high-energy photons, and the electrically grounded potential on the flow channel terminates charged species.

A schematic illustration of the oxygen-radical generator is shown in Additional file 1: Figure S8a. Vanillin (1.0 mM, 2.5 mM, and 5.0 mM) dissolved in 0.25% acetonitrile solution (3.0 mL) was irradiated with oxygen radical using the oxygen-radical generator. A fixed distance of 1 cm was used between the slit exit of the radical generator and the surface of the liquid suspension. The suspension samples in Petri dishes (30-mm diameter) were placed on an automated stage for uniform treatment of the solution due to the shape of the radical exit (0.5 × 16 mm). The speed of the automated stage was set at 4 mm/s, and a plastic chamber was covered to avoid mixing with ambient air.

### Yeast strain, growth, and ethanol production

*S. cerevisiae* S288c was obtained from NITE Biological Resource Center (Tokyo, Japan) and cultured in liquid yeast-extract–peptone–dextrose (YPD) medium (10 g/L yeast extract, 20 g/L peptone, and 20 g/L glucose) containing 1.0 mM, 2.5 mM, and 5.0 mM vanillin with shaking at 100 rpm at 28 °C for up to 16 h. Cell growth in the presence of vanillin with or without oxygen-radical irradiation was monitored by measuring the optical density at 600 nm. Ethanol in the culture supernatant was measured using an ethanol assay kit (Megazyme International, Bray, Ireland).

### Alkaline pretreatment and oxygen-radical irradiation of rice straw

Prior to alkaline pretreatment, rice straw was milled to a particle size of 1 mm and then washed and dried at 45 °C for 24 h, after which the dried rice straw (20 g) was suspended in 400 mL of 1 N NaOH solution (at 5% (w/v) solid loading in 1 L Erlenmeyer flask), and two-step alkaline pretreatment was applied at 37 °C for 24 h with shaking at 100 rpm, followed by autoclaving at 120 °C for 60 min. The prepared alkaline-pretreated slurry was neutralized at pH 6 with 6 N HCl. Glycine (at a final concentration of 50 μM), which is not affected by oxygen-radical treatment [31], was added to the neutralized rice straw slurry as an internal standard for quantitative analysis using GC–MS. Oxygen radical was then used to sequentially irradiate the slurry, as described.

### Chemical composition analysis

The cellulose, hemicellulose, and lignin compositions of native rice straw, and the remaining solids filtered from the alkaline-pretreated rice straw slurry with or without oxygen-radical treatment were analysed according to the National Renewable Energy Laboratory (NREL) protocol [59]. Samples (300 mg) were mixed into 3 mL of 72% (w/w) sulfuric acid at 30 °C for 60 min. Then, the sulfuric acid was diluted to 4.0% by adding 84 mL deionized water. The mixture was incubated at 121 °C for 60 min. Then the mixture was cooled to room temperature, and the residue was removed by filtration and the supernatant was collected and determined by a Prominence reducing-sugar high-performance liquid chromatography (HPLC) analytical system (Shimadzu, Kyoto, Japan) to measure the monomeric sugar content including glucose, xylose, arabinose, galactose, and mannose. The concentration of cellulose and hemicellulose was calculated according to the monomeric sugar content.

Moreover, the acid-soluble lignin (ASL) content in the liquid was detected using a UV–visible spectrophotometer. The residue was used to determine the acid-insoluble lignin (AIL) content with a muffle furnace at 575 ± 25 °C for 24 h. Ash and total solids were also determined using the muffle furnace and a hot-air oven, respectively [60, 61].

### Saccharification of alkaline-pretreated rice straw

Alkaline-pretreated rice straw slurry (with or without oxygen-radical treatment) was hydrolysed by cellulase from *A. niger* (Tokyo Chemical Industry Co., Ltd.) with enzyme loading at 6.0 mg of protein per gramme of cellulose. Saccharification proceeded at 37 °C for 48 h with shaking at 120 rpm. The hydrolysate was separated by filtration, and the filtrate was sterilized using a 0.22 µm polyethersulfone (PES) syringe filter and added to the yeast extract (at a final concentration of 1%) and peptone (at a final concentration of 2%) to culture yeast cells for 48 h. A schematic illustration of yeast growth and ethanol production using the alkaline-pretreated rice straw slurry with or without oxygen-radical and cellulase treatments following filter sterilization is shown in Additional file 1: Figure S8b.

### Analytical methods

Vanillin solution (10 μL) treated with or without oxygen radical and the hydrolysate (10 μL) obtained from alkaline-pretreated rice straw with or without oxygen-radical and cellulase treatments following filter sterilization were analysed using an Acuity ultra-performance liquid chromatography (Waters, Milford, MA) equipped with an ADME-HR S5 column (150 × 4.6 mm i.d. × 5 µm pore size; Osaka Soda, Osaka, Japan). Vanillin solutions and the hydrolysates (500 μL) were lyophilized, trimethylsilylated using 50 μL of *N*-methyl-*N*-trimethylsilyltrifluoroacetamide (Wako Pure Chemical Industries), and analysed using gas chromatography–mass spectrometry (GC–MS; GCMS-QP2010; Shimadzu, Kyoto, Japan) on a system equipped with a J&W DB-5MS capillary column (30 m × 0.25 mm i.d. × 0.25 μm thickness; Agilent Technologies, Santa Clara, CA) [62]. Glycine (at a final concentration of 50 μM) was used an internal standard for quantitative analysis using GC–MS. We determined the reducing sugar content in the hydrolysates obtained from alkaline-pretreated rice straw with or without oxygen-radical and cellulase treatments following filter sterilization. Reducing-sugars in the filtrates (10 μL) obtained from alkaline-pretreated rice straw with or without oxygen-radical and cellulase treatments following filter sterilization were also determined by monitoring post-column derivatized reducing sugars that were separated using a Prominence reducing-sugar HPLC analytical system equipped with a fluorescence detector. The supernatant was separated on a Shim-pack 4.0 × 250-mm ISA-07/S2504 column (Shimadzu) with a linear gradient of 0.1 M potassium borate buffer (pH 8.0) and 0.4 M potassium borate buffer (pH 9.0) for 120 min at a flow rate of 0.6 mL min^−1^ [30, 63, 64].

## Supplementary information

**Additional file 1: Figure S1.** Treatment-time-dependent conversion of vanillin (5.0 mM) and the production of reactants monitored by HPLC. Identified reaction products are marked by arrows with numbers and shown in Table 1. **Figure S2.** MS analysis of the trimethylsilyl (TMS) derivatives among the reaction products generated from vanillin by oxygen-radical treatment. Each number indicates the GC peaks shown in Fig. 1a and Table 1. **Figure S3.** Vanillin oxidation, monooxygenation, demethoxylation, decarbonylation, and aromatic-ring fission by oxygen-radical irradiation. Each number indicates the GC peaks shown in Fig. 1b and Table 1. **Figure S4.** Effects of vanillin degradation products on the growth of *S. cerevisiae*. The yeast was grown in YPD medium supplemented with 2.5 mM vanillin degradation products, such as vanillic acid, protocatechuic aldehyde, protocatechuic acid, methoxyhydroquinone, 3,4-dihydroxy-5-methoxybenzaldehyde, and oxalic acid. Yeast growth was monitored by measuring optical density at 600 nm. Error bars represent the mean ± standard error of the mean of three independent experiments. **Figure S5.** Effects of several compounds generated from alkaline-pretreated rice straw with or without oxygen-radical treatment on the growth of *S. cerevisiae*. The yeast was grown in YPD medium supplemented with 2.5 mM *p*-coumaric acid, *t*-ferulic acid, lactic acid, and furfural. Yeast growth was monitored by measuring optical density at 600 nm. Error bars represent the mean ± standard error of the mean of three independent experiments. **Figure S6.** The content of glucose, cellobiose, cellotriose, and xylose in alkaline-pretreated rice straw slurry with or without oxygen-radical and cellulase treatments. Sugars released from alkaline-pretreated rice straw after enzymatic hydrolysis using commercially available cellulase from *A. niger* were quantified by reducing-sugar HPLC. Data are presented as the mean ± standard deviation of three experiments. **Figure S7.** Effects of oxygen-radical treatment of glucose on the growth of *S. cerevisiae*. (a) TLC analysis of 10, 25, and 50 mM glucose solutions irradiated with oxygen-radical treatment for 0 min (−) and 20 min (+). The procedure of TLC analysis was described previously [30]. (b) The yeast was grown in 50 mM glucose medium containing yeast extract (at a final concentration of 1%) and peptone (at a final concentration of 2%) with or without oxygen-radical treatment. Yeast growth was monitored by measuring optical density at 600 nm. Error bars represent the mean ± standard error of the mean of three independent experiments. **Figure S8.** Schematic diagram of sample preparation for oxygen-radical treatment. (a) Radical-treatment conditions were optimized to obtain maximal atomic oxygen [O (^3^P_*j*_)]. All samples were suspended in 3-mL solutions, and a fixed distance of 1 cm was used between the slit exit of the radical generator and the surface of the liquid suspension. (b) Flow chart of sample preparation used in this study for ethanol production by *S. cerevisiae* using alkaline-pretreated rice straw slurry with or without oxygen-radical and cellulase treatments.

## Data Availability

All data generated or analysed during this study are included in this published article.

## Abbreviations

NTAP: non-thermal atmospheric pressure plasma
YPD: yeast-extract–peptone–dextrose
GC–MS: gas chromatography–mass spectrometry
HPLC: high-performance liquid chromatography
CBHs: cellobiohydrolases

## References

[1] Luo L, van der Voet E, Huppes G (2010). Biorefining of lignocellulosic feedstock-technical, economic and environmental considerations. Bioresour Technol.

[2] Zeng Y, Zhao S, Yang S, Ding SY (2014). Lignin plays a negative role in the biochemical process for producing lignocellulosic biofuels. Curr Opin Biotechnol.

[3] Simmons BA, Loqué D, Ralph J (2010). Advances in modifying lignin for enhanced biofuel production. Curr opin Plant biol.

[4] Anterola AM, Lewis NG (2002). Trends in lignin modification: a comprehensive analysis of the effects of genetic manipulations/mutations on lignification and vascular integrity. Phytochemistry.

[5] Chen H, Qui W (2010). Key technologies for bioethanol production from lignocellulose. Biotechnol Adv.

[6] Alvira P, Tomás-Pejó E, Ballesteros M, Negro MJ (2010). Pretreatment technologies for an efficient bioethanol production process based on enzymatic hydrolysis: a review. Bioresour Technol.

[7] Singh S, Cheng G, Sathitsuksanoh N, Wu D, Varanasi P, George A, Balan V, Gao X, Kumar R, Dale BE, Wyman CE, Simmons BA (2015). Comparison of different biomass pretreatment techniques and their impact on chemistry and structure. Front Energy Res.

[8] Budarin VL, Clark JH, Lanigan BA, Shuttleworth P, Macquarrie DJ (2010). Microwave assisted decomposition of cellulose: a new thermochemical route for biomass exploitation. Bioresour Technol.

[9] Galia A, Schiavo B, Antonetti C, Galletti AM, Interrante L, Lessi M, Scialdone O, Valenti MG (2015). Autohydrolysis pretreatment of *Arundo donax*: a comparison between microwave-assisted batch and fast heating rate flow-through reaction systems. Biotechnol Biofuels.

[10] Koda S, Taguchi K, Futamura K (2011). Effects of frequency and a radical scavenger on ultrasonic degradation of water-soluble polymers. Ultrason Sonochem.

[11] Tian D, Chandra RP, Lee JS, Lu C, Saddler JN (2017). A comparison of various lignin-extraction methods to enhance the accessibility and ease of enzymatic hydrolysis of the cellulosic component of steam-pretreated poplar. Biotechnol Biofuels.

[12] Wen P, Zhang T, Wang J, Lian Z, Zhang J (2019). Production of xylooligosaccharides and monosaccharides from poplar by a two-step acetic acid and peroxide/acetic acid pretreatment. Biotechnol Biofuels.

[13] Du B, Sharma LN, Becker C, Chen SF, Mowery RA, van Walsum GP, Chambliss CK (2010). Effect of varying feedstock-pretreatment chemistry combinations on the formation and accumulation of potentially inhibitory degradation products in biomass hydrolysates. Biotechnol Bioeng.

[14] Kim Y, Kreke T, Hendrickson R, Parenti J, Ladisch MR (2013). Fractionation of cellulase and fermentation inhibitors from steam pretreated mixed hardwood. Bioresour Technol.

[15] Almeida JRM, Modig T, Petersson A, Hähn-Hägerdal B, Lidén G, Gorwa-Grauslund MF (2007). Increased tolerance and conversion of inhibitors in lignocellulosic hydrolysates by *Saccharomyces cerevisiae*. J Chem Technol Biotechnol.

[16] Heer D, Sauer U (2008). Identification of furfural as a key toxin in lignocellulosic hydrolysates and evolution of a tolerant yeast strain. Microb Biotechnol.

[17] Palmqvist E, Hahn-Hägerdal B (2000). Fermentation of lignocellulosic hydrolysates. I: inhibition and detoxification. Bioresour Technol.

[18] Pampulha ME, Loureiro-Dias MC (2000). Energetics of the effect of acetic acid on growth of *Saccharomyces cerevisiae*. FEMS Microbiol Lett.

[19] Helle S, Cameron D, Lam J, White B, Duff S (2003). Effect of inhibitory compounds found in biomass hydrolysates on growth and xylose fermentation by a genetically engineered strain of *S. cerevisiae*. Enzyme Microb Technol.

[20] Klinke HB, Thomsen AB, Ahring BK (2004). Inhibition of ethanol-producing yeast and bacteria by degradation products produced during pre-treatment of biomass. Appl Microbiol Biotechnol.

[21] Zhang Y, Xia C, Lu M, Tu M (2018). Effect of overliming and activated carbon detoxification on inhibitors removal and butanol fermentation of poplar prehydrolysates. Biotechnol Biofuels.

[22] Maddox IS, Murray AE (1983). Production of *n*-butanol by fermentation of wood hydrolysate. Biotechnol Lett.

[23] Lee JM, Venditti RA, Jameel H, Kenealy WR (2011). Detoxification of woody hydrolyzates with activated carbon for bioconversion to ethanol by the thermophilic anaerobic bacterium *Thermoanaerobacterium saccharolyticum*. Biomass Bioenergy.

[24] Kim Y, Ximenes E, Mosier NS, Ladisch MR (2011). Soluble inhibitors/deactivators of cellulase enzymes from lignocellulosic biomass. Enzyme Microb Technol.

[25] Larsson S, Reimann A, Nilvebrant NO, Jonsson LJ (1999). Comparison of different methods for the detoxification of lignocellulose hydrolyzates of spruce. Appl Biochem Biotechnol.

[26] Inui H, Takeda K, Kondo H, Ishikawa K, Sekine M, Kano H, Yoshida N, Hori M (2010). Measurement of hydrogen radical density and its impact on reduction of copper oxide in atmospheric pressure remote plasma using H_2_ and Ar mixture gases. Appl Phys Express.

[27] Iwasaki M, Matsudaira Y, Takeda K, Ito M, Miyamoto E, Yara T, Uehara T (2008). Roles of oxidizing species in a non-equilibrium atmospheric pressure pulsed remote O_2_/N_2_ plasma glass cleaning process. J Appl Phys.

[28] Hashizume H, Ohta T, Fengdong J, Takeda K, Ishikawa K, Hori M, Ito M (2013). Inactivation effects of neutral reactive-oxygen species on *Penicillium digitatum* spores using non-equilibrium atmospheric-pressure oxygen radical source. Appl Phys Lett.

[29] Ito M, Oh JS, Ohta T, Shiratani M, Hori M (2018). Current status and future prospects of agricultural applications using atmospheric-pressure plasma technologies. Plasma Process Polym.

[30] Sakai K, Kojiya S, Kamijo J, Tanaka Y, Tanaka K, Maebayashi M, Oh JS, Ito M, Hori M, Shimizu M, Kato M (2017). Oxygen-radical pretreatment promotes cellulose degradation by cellulolytic enzymes. Biotechnol Biofuels.

[31] Takai E, Kitamura T, Kuwabara J, Ikawa S, Yoshizawa S, Shiraki K, Kawasaki H, Arakawa R, Kitano K (2014). Chemical modification of amino acids by atmospheric-pressure cold plasma in aqueous solution. J Phys D Appl Phys.

[32] Takai E, Kitano K, Kuwabara J, Shiraki K (2012). Protein Inactivation by low-temperature atmospheric pressure plasma in aqueous solution. Plasma Process Polym.

[33] Stadtman ER, Levine RL (2003). Free radical-mediated oxidation of free amino acids and amino acid residues in proteins. Amino Acids.

[34] Asandulesa M, Topala I, Pohoata V, Legrand YM, Dobromir M, Totolin M, Dumitrascu N (2013). Chemically polymerization mechanism of aromatic compounds under atmospheric pressure plasma conditions. Plasma Process Polym.

[35] Sein MM, Zedda M, Tuerk J, Schmidt TC, Golloch A, von Sonntag C (2008). Oxidation of diclofenac with ozone in aqueous solution. Environ Sci Technol.

[36] Tentscher PR, Bourgin M, von Gunten U (2018). Ozonation of para-substituted phenolic compounds yields p-benzoquinones, other cyclic α,β-unsaturated ketones, and substituted catechols. Environ Sci Technol.

[37] Figueirêdo MB, Deuss PJ, Venderbosch RH, Heeres HJ (2019). Valorization of pyrolysis liquids: ozonation of the pyrolytic lignin fraction and model components. ACS Sustain Chem Eng.

[38] Endo A, Nakamura T, Ando A, Tokuyasu K, Shima J (2008). Genome-wide screening of the genes required for tolerance to vanillin, which is a potential inhibitor of bioethanol fermentation, in *Saccharomyces cerevisiae*. Biotechnol Biofuels.

[39] Iwaki A, Ohnuki S, Suga Y, Izawa S, Ohya Y (2013). Vanillin inhibits translation and induces messenger ribonucleoprotein (mRNP) granule formation in *Saccharomyces cerevisiae*: application and validation of high-content, image-based profiling. PLoS ONE.

[40] Nguyen TT, Iwaki A, Ohya Y, Izawa S (2014). Vanillin causes the activation of Yap1 and mitochondrial fragmentation in *Saccharomyces cerevisiae*. J Biosci Bioeng.

[41] Kim JH, Lee HO, Cho YJ, Kim J, Chun J, Choi J, Lee Y, Jung WH (2014). Vanillin derivative causes mitochondrial dysfunction and triggers oxidative stress in *Cryptococcus neoformans*. PLoS ONE.

[42] Wang X, Liang Z, Hou J, Bao X, Shen Y (2016). Identification and functional evaluation of the reductases and dehydrogenases from *Saccharomyces cerevisiae* involved in vanillin resistance. BMC Biotechnol.

[43] Pereira FB, Guimarães PMR, Gomes DG, Mira NP, Teixeira MC, Sá-Correia I, Domingues L (2011). Identification of candidate genes for yeast engineering to improve bioethanol production in very high gravity and lignocellulosic biomass industrial fermentations. Biotechnol Biofuels.

[44] Adeboye PT, Bettiga M, Aldaeus F, Larsson PT, Olsson L (2015). Catabolism of coniferyl aldehyde, ferulic acid and *p*-coumaric acid by *Saccharomyces cerevisiae* yields less toxic products. Microb Cell Fact.

[45] Adeboye PT, Olsson L, Bettiga M (2016). A coniferyl aldehyde dehydrogenase gene from *Pseudomonas* sp. strain HR199 enhances the conversion of coniferyl aldehyde by *Saccharomyces cerevisiae*. Bioresour Technol.

[46] Klinke HB, Thomsen AB, Ahring BK (2001). Potential inhibitors from wet oxidation of wheat straw and their effect on growth and ethanol production by *Thermoanaerobacter mathranii*. Appl Microbiol Biotechnol.

[47] Li J, Shi S, Adhikaria S, Tu M (2017). Inhibition effect of aromatic aldehydes on butanol fermentation by *Clostridium acetobutylicum*. RSC Adv.

[48] Cho DH, Lee YJ, Um Y, Sang BI, Kim YH (2009). Detoxification of model phenolic compounds in lignocellulosic hydrolysates with peroxidase for butanol production from *Clostridium beijerinckii*. Appl Microbiol Biotechnol.

[49] Liu Y, Geng Y, Zhou Q, Yuan W (2018). The effects of syringaldehyde and vanillin on butyric acid production by fermentation using *Clostridium tyrobutyricum*. Bioresources.

[50] Cao GL, Ren NQ, Wang AJ, Guo WQ, Xu JF, Liu BF (2010). Effect of lignocellulose-derived inhibitors on growth and hydrogen production by *Thermoanaerobacterium thermosaccharolyticum* W16. Int J Hydrog Energy.

[51] Zhang S, Winestrand S, Guo X, Chen L, Hong F, Jönsson LJ (2014). Effects of aromatic compounds on the production of bacterial nanocellulose by *Gluconacetobacter xylinus*. Microb Cell Fact.

[52] Wang L (2013). Effect of selected aldehydes found in the corncob hemicellulose hydrolysate on the growth and xylitol fermentation of *Candida tropicalis*. Biotechnol Prog.

[53] Baranowski JD, Davidson PM, Nagel CW, Branen AL (1980). Inhibition of *Saccharomyces cerevisiae* by naturally occurring hydroxycinnamates. J Food Sci.

[54] Adav SS, Li AA, Manavalan A, Punt P, Sze SK (2010). Quantitative iTRAQ secretome analysis of *Aspergillus niger* reveals novel hydrolytic enzymes. J Proteome Res.

[55] Florencio C, Cunha FM, Badino AC, Farinas CS, Ximenes E, Ladisch MR (2016). Secretome analysis of *Trichoderma reesei* and *Aspergillus niger* cultivated by submerged and sequential fermentation processes: enzyme production for sugarcane bagasse hydrolysis. Enzyme Microb Technol.

[56] Li Y, Qi B, Wan Y (2014). Inhibitory effect of vanillin on cellulase activity in hydrolysis of cellulosic biomass. Bioresour Technol.

[57] Qin L, Li WC, Liu L, Zhu JQ, Li X, Li BZ, Yuan YJ (2016). Inhibition of lignin-derived phenolic compounds to cellulase. Biotechnol Biofuels.

[58] Caspeta L, Castillo T, Nielsen J (2015). Modifying yeast tolerance to inhibitory conditions of ethanol production processes. Front Bioeng Biotechnol.

[59] Sluiter A, Hames B, Ruiz R, Scarlata C, Sluiter J, Templeton D, Crocker D (2008). Determination of structural carbohydrates and lignin in biomass, NREL/TP-510-42618.

[60] Sluiter A, Hames B, Ruiz R, Scarlata C, Sluiter J, Templeton D (2008). Determination of ash in biomass, NREL/TP-510-42622.

[61] Sluiter A, Hames B, Hyman D, Payne C, Ruiz R, Scarlata C, Sluiter J, Templeton D, Wolfe J (2008). Determination of total solids in biomass and total dissolved solids in liquid process samples, NREL/TP-510-42621.

[62] Sakai K, Matsuzaki F, Wise L, Sakai Y, Jindou S, Ichinose H, Takaya N, Kato M, Wariishi H, Shimizu M (2018). Biochemical characterization of CYP505D6, a self-sufficient cytochrome P450 from the white-rot fungus *Phanerochaete chrysosporium*. Appl Environ Microbiol.

[63] Sakai K, Mochizuki M, Yamada M, Shinzawa Y, Minezawa M, Kimoto S, Murata S, Kaneko Y, Ishihara S, Jindou S, Kobayashi T, Kato M, Shimizu M (2017). Biochemical characterization of thermostable β-1,4-mannanase belonging to the glycoside hydrolase family 134 from *Aspergillus oryzae*. Appl Microbiol Biotechnol.

[64] Shimizu M, Kaneko Y, Ishihara S, Mochizuki M, Sakai K, Yamada M, Murata S, Itoh E, Yamamoto T, Sugimura Y, Hirano T, Takaya N, Kobayashi T, Kato M (2015). Novel β-1,4-mannanase belonging to a new glycoside hydrolase family in *Aspergillus nidulans*. J Biol Chem.

