# Supplementary material for: Oxygen radical based on non-thermal atmospheric pressure plasma alleviates lignin-derived phenolic toxicity in yeast
Source: Biotechnol Biofuels. 2020 Jan 28;13:18. doi: 10.1186/s13068-020-1655-9 (PMC6988259; doi:10.1186/s13068-020-1655-9)
Supplement: Supplementary file 1 — Additional file 1: Figure S1. Treatment-time-dependent conversion of vanillin (5.0 mM) and the production of reactants monitored by HPLC. Identified reaction products are marked by arrows with numbers and shown in Table 1. Figure S2. MS analysis of the trimethylsilyl (TMS) derivatives among the reaction products generated from vanillin by oxygen-radical treatment. Each number indicates the GC peaks shown in Fig. 1a and Table 1. Figure S3. Vanillin oxidation, monooxygenation, demethoxylation, decarbonylation, and aromatic-ring fission by oxygen-radical irradiation. Each number indicates the GC peaks shown in Fig. 1b and Table 1. Figure S4. Effects of vanillin degradation products on the growth of S. cerevisiae. The yeast was grown in YPD medium supplemented with 2.5 mM vanillin degradation products, such as vanillic acid, protocatechuic aldehyde, protocatechuic acid, methoxyhydroquinone, 3,4-dihydroxy-5-methoxybenzaldehyde, and oxalic acid. Yeast growth was monitored by measuring optical density at 600 nm. Error bars represent the mean ± standard error of the mean of three independent experiments. Figure S5. Effects of several compounds generated from alkaline-pretreated rice straw with or without oxygen-radical treatment on the growth of S. cerevisiae. The yeast was grown in YPD medium supplemented with 2.5 mM p-coumaric acid, t-ferulic acid, lactic acid, and furfural. Yeast growth was monitored by measuring optical density at 600 nm. Error bars represent the mean ± standard error of the mean of three independent experiments. Figure S6. The content of glucose, cellobiose, cellotriose, and xylose in alkaline-pretreated rice straw slurry with or without oxygen-radical and cellulase treatments. Sugars released from alkaline-pretreated rice straw after enzymatic hydrolysis using commercially available cellulase from A. niger were quantified by reducing-sugar HPLC. Data are presented as the mean ± standard deviation of three experiments. Figure S7. Effects of oxygen-radi [file 13068_2020_1655_MOESM1_ESM.docx]

**Additional file 1**

**Oxygen-radical based on non-thermal atmospheric pressure plasma alleviates lignin-derived phenolics toxicity in yeast**

Shou Ito^1†^, Kiyota Sakai^1†^, Vladislav Gamaleev^2^, Masafumi Ito^2^, Masaru Hori^3^, Masashi Kato^1^, Motoyuki Shimizu^1^*

^1^Faculty of Agriculture, Meijo University, Nagoya, Aichi 468-8502, Japan

^2^Faculty of Science and Technology, Meijo University, Nagoya, Aichi 468-8502, Japan

^3^Center for Low-temperature Plasma Sciences, Nagoya University, Nagoya, Aichi 464-8603, Japan

* Corresponding author.

Tel/Fax: +81-52-838-2445

E-mail: moshimi@meijo-u.ac.jp (M. Shimizu).

^†^ These authors contributed equally to this work.

**Fig. S1** **Treatment-time-dependent conversion of vanillin (5.0 mM) and the production of reactants monitored by HPLC.** Identified reaction products are marked by arrows with numbers and shown in Table 1.

**Fig. S2 MS analysis of the trimethylsilyl (TMS) derivatives among the reaction products generated from vanillin by oxygen-radical treatment.** Each number indicates the GC peaks shown in Fig. 1a and Table 1.


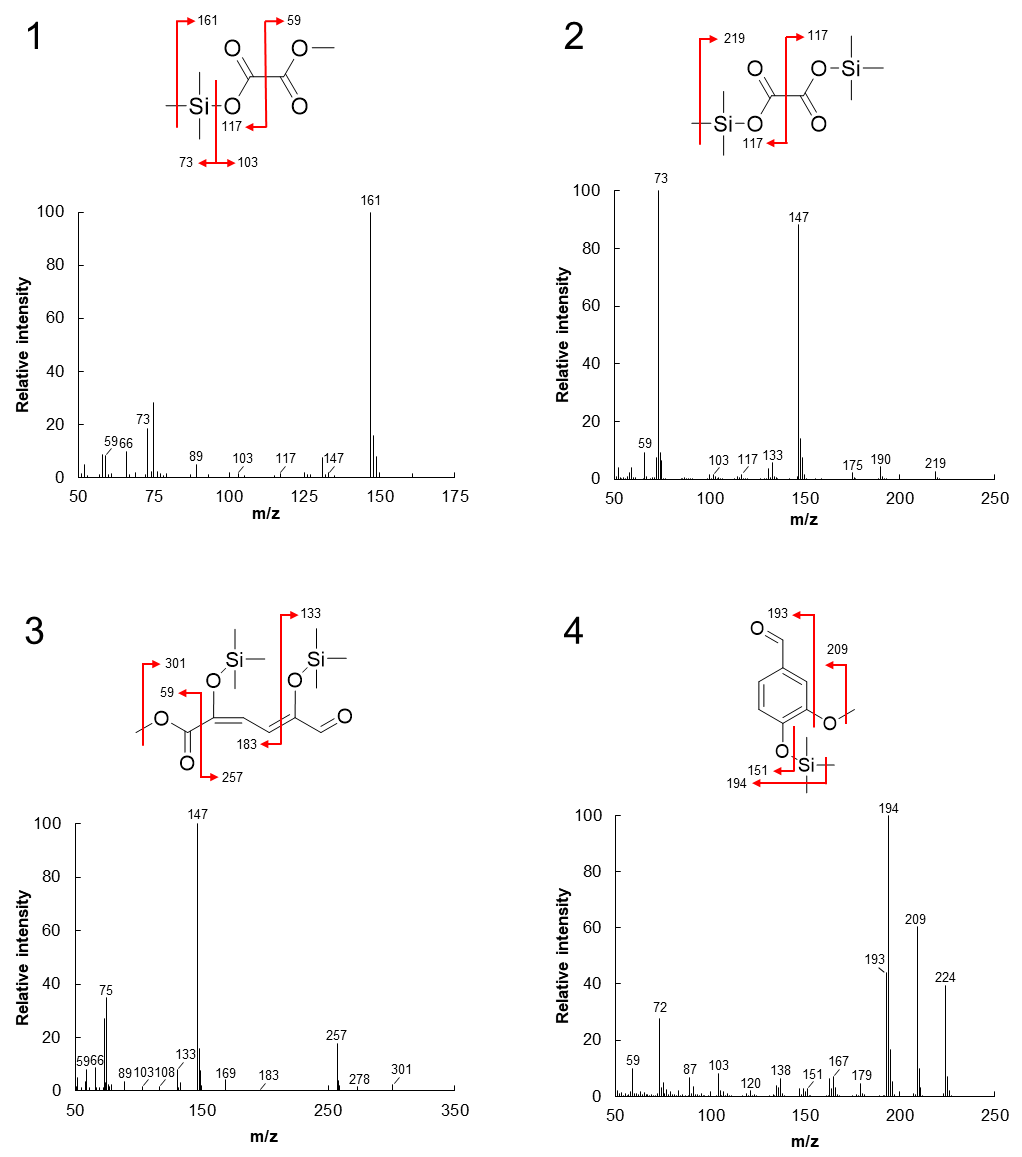


**
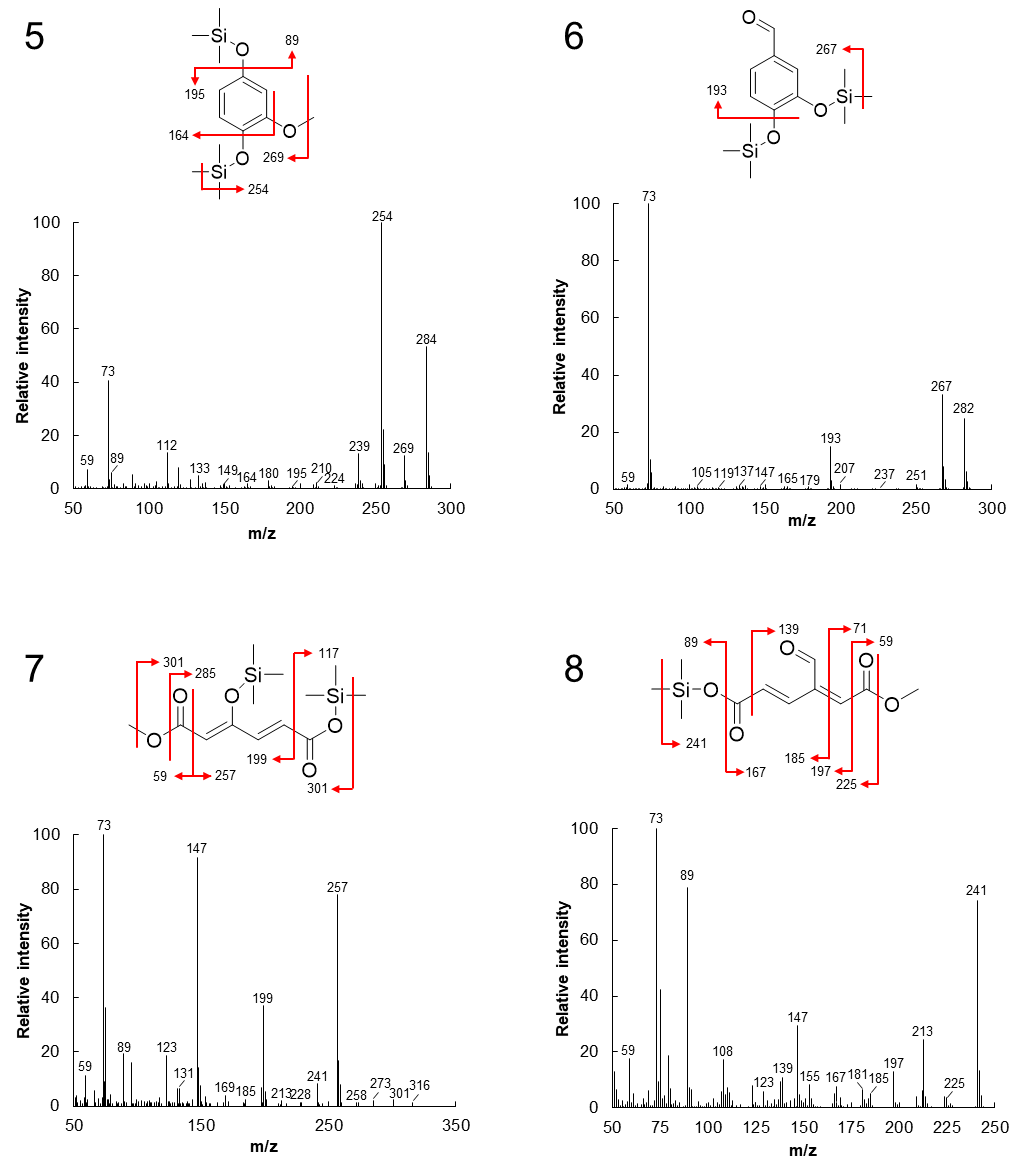
**


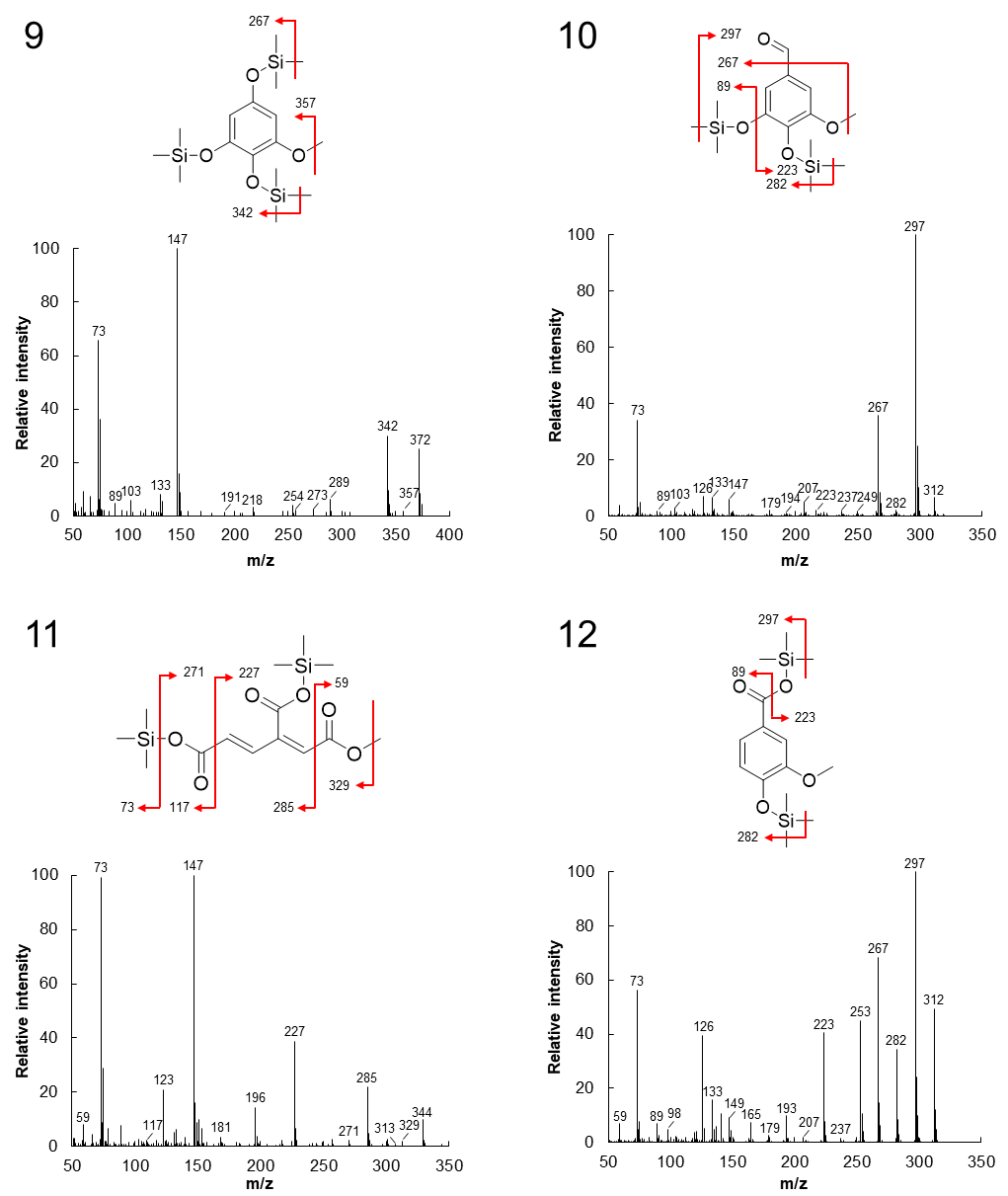


**
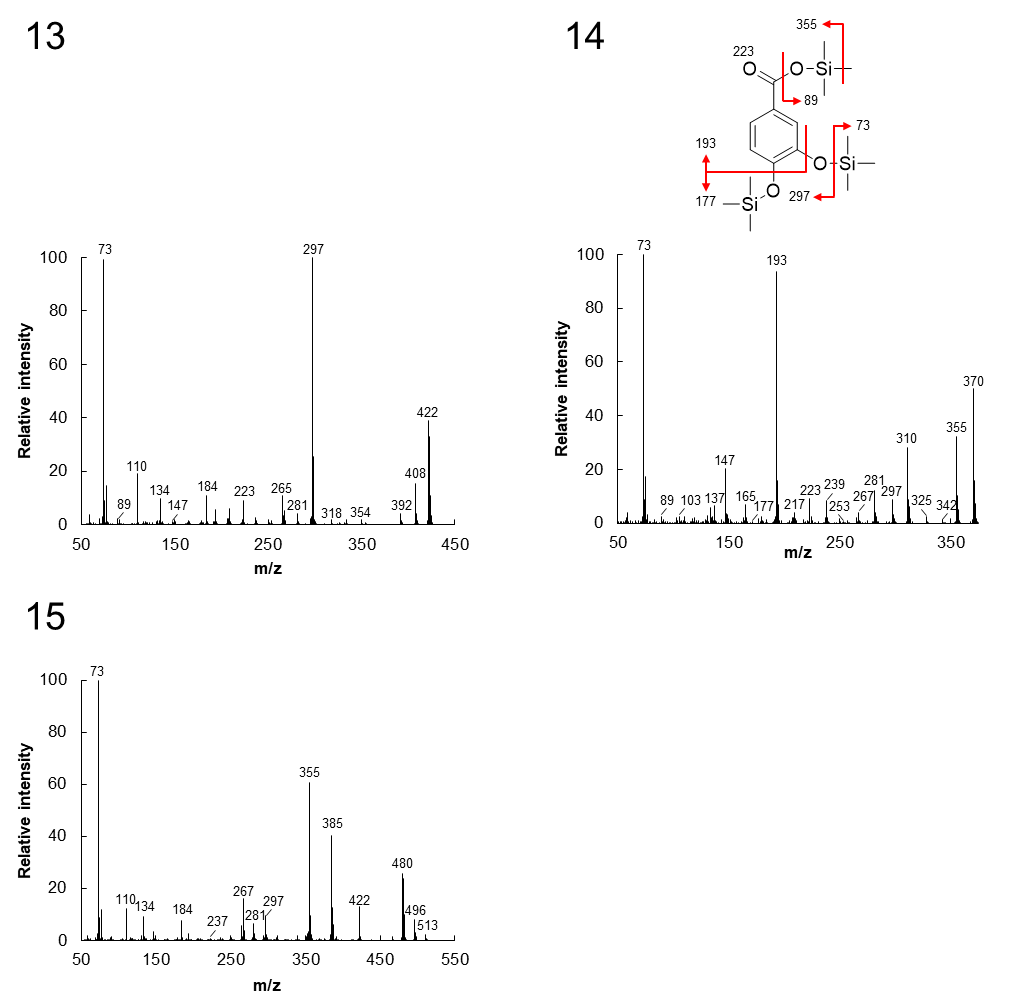
**

**Fig. S3 Vanillin oxidation, monooxygenation, demethoxylation, decarbonylation, and aromatic-ring fission by oxygen-radical irradiation.** Each number indicates the GC peaks shown in Fig. 1b and Table 1.

**Fig. S4 Effects of vanillin degradation products on the growth of *S. cerevisiae*.** The yeast was grown in YPD medium supplemented with 2.5 mM vanillin degradation products, such as vanillic acid, protocatechuic aldehyde, protocatechuic acid, methoxyhydroquinone, 3,4-dihydroxy-5-methoxybenzaldehyde, and oxalic acid. Yeast growth was monitored by measuring optical density at 600 nm. Error bars represent the mean ± standard error of the mean of three independent experiments.

**Fig. S5 Effects of several compounds generated from alkaline-pretreated rice straw with or without oxygen-radical treatment on the growth of *S. cerevisiae*.** The yeast was grown in YPD medium supplemented with 2.5 mM *p*-coumaric acid, *t*-ferulic acid, lactic acid, and furfural. Yeast growth was monitored by measuring optical density at 600 nm. Error bars represent the mean ± standard error of the mean of three independent experiments.

**Fig. S6 The content of glucose, cellobiose, cellotriose, and xylose in alkaline-pretreated rice straw slurry with or without oxygen-radical and cellulase treatments.** Sugars released from alkaline-pretreated rice straw after enzymatic hydrolysis using commercially available cellulase from *A. niger* were quantified by reducing-sugar HPLC. Data are presented as the mean ± standard deviation of three experiments.

**Fig. S7 Effects of oxygen-radical treatment of glucose on the growth of *S. cerevisiae*.** (a) TLC analysis of 10, 25, and 50 mM glucose solutions irradiated with oxygen-radical treatment for 0 min (-) and 20 min (+). The procedure of TLC analysis was described previously [30]. (b) The yeast was grown in 50 mM glucose medium containing yeast extract (at a final concentration of 1%) and peptone (at a final concentration of 2%) with or without oxygen-radical treatment. Yeast growth was monitored by measuring optical density at 600 nm. Error bars represent the mean ± standard error of the mean of three independent experiments.

b

a


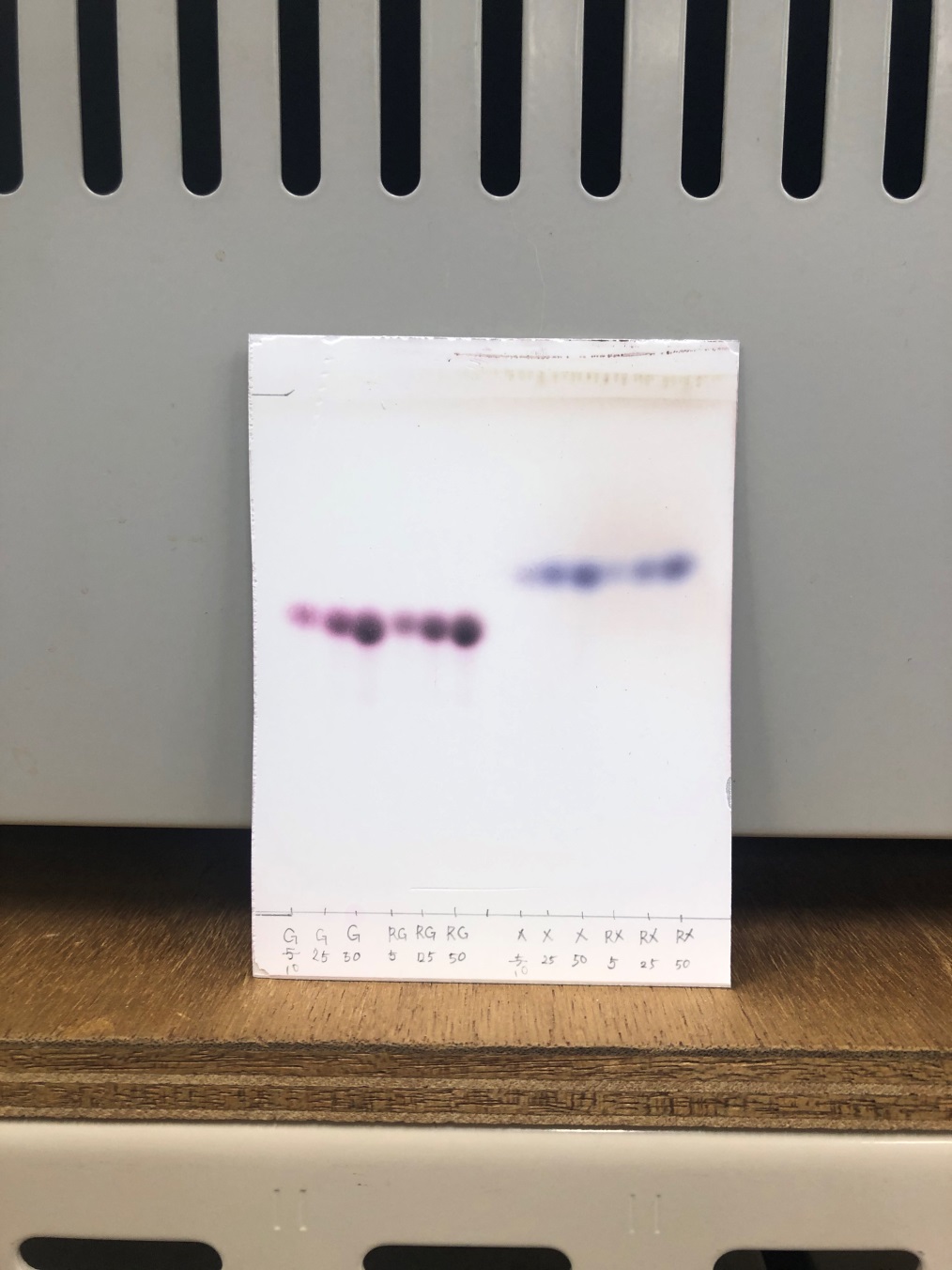


10

25

50

25

50

+

Glucose

-

10

**Fig. S8 Schematic diagram of sample preparation for oxygen-radical treatment.** (a) Radical-treatment conditions were optimized to obtain maximal atomic oxygen [O (^3^P*_j_*)]. All samples were suspended in 3-mL solutions, and a fixed distance of 1 cm was used between the slit exit of the radical generator and the surface of the liquid suspension. (b) Flow chart of sample preparation used in this study for ethanol production by *S. cerevisiae* using alkaline-pretreated rice straw slurry with or without oxygen-radical and cellulase treatments.

b

a
